# Physical restraint use among nursing home residents: A comparison of two data collection methods

**DOI:** 10.1186/1472-6955-3-5

**Published:** 2004-10-15

**Authors:** Danielle Laurin, Philippe Voyer, René Verreault, Pierre J Durand

**Affiliations:** 1 Laval University Geriatric Research Unit, Quebec, CANADA; 2 Faculty of Pharmacy, Laval University, Quebec, CANADA; 3 Faculty of Nursing Sciences, Laval University, Quebec, CANADA; 4 Department of Social and Preventive Medicine, Faculty of Medicine, Laval University, Quebec, CANADA

## Abstract

**Background:**

In view of the issues surrounding physical restraint use, it is important to have a method of measurement as valid and reliable as possible. We determined the sensitivity and specificity of physical restraint use a) reported by nursing staff and b) reviewed from medical and nursing records in nursing home settings, by comparing these methods with direct observation.

**Methods:**

We sampled eight care units in skilled nursing homes, seven care units in nursing homes and one long-term care unit in a hospital, from eight facilities which included 28 nurses and 377 residents. Physical restraint use was assessed the day following three periods of direct observation by two different means: interview with one or several members of the regular nursing staff, and review of medical and nursing records. Sensitivity and specificity values were calculated according to 2-by-2 contingency tables. Differences between the methods were assessed using the phi coefficient. Other information collected included: demographic characteristics, disruptive behaviors, body alignment problems, cognitive and functional skills.

**Results:**

Compared to direct observation (gold standard), reported restraint use by nursing staff yielded a sensitivity of 87.4% at a specificity of 93.7% (phi = 0.84). When data was reviewed from subjects' medical and nursing records, sensitivity was reduced to 74.8%, and specificity to 86.3% (phi = 0.54). Justifications for restraint use including risk for falls, agitation, body alignment problems and aggressiveness were associated with the use of physical restraints.

**Conclusions:**

The interview of nursing staff and the review of medical and nursing records are both valid and reliable techniques for measuring physical restraint use among nursing home residents. Higher sensitivity and specificity values were achieved when nursing staff was interviewed as compared to reviewing medical records. This study suggests that the interview of nursing staff is a more reliable method of data collection.

## Background

Nursing homes have the mandate to offer care settings to frail dependent older individuals. However, a renewed emphasis has emerged over the past decades to become more than just a home for older people [1]. A growing number of facilities are actually striving to preserve residents' sense of control and dignity in order to achieve the highest level of well-being [2,3]. This new way of thinking is based upon values of respect of autonomy and freedom for older persons in various ways such as the resident's right to take risks or to make his/her own choices [4].

Although predominantly intended as protective devices, physical restraints in nursing homes are being denunciated as measures that go conversely with the aforementioned principles [5]. Justifications for controlling confusion, agitated and aggressive behaviors are being questioned [6-8] and beneficial effects of physical restraints on falls and injuries, incontinence, muscle atrophy and quality of life challenged [8-15]. Moreover, physical restraints have been associated with cognitive impairment, nosocomial infections, pressure sores and death [10,16-19].

According to the literature, the overall prevalence of restraint use in nursing homes ranges between 4 and 68% [5,20]. This wide variation may be explained by definitions of physical restraints used, study sample sizes, characteristics of care settings, and residents' characteristics and cognitive status. Another explanation could be the choice of techniques of data collection. Several methods have been used alone or in combination for the measurement of physical restraint use [20-22]: direct observation, survey or interview of nursing staff, review of medical and nursing records and, when the cognitive status allows it, interview with residents themselves. In view of the consequential issues surrounding the use of physical restraints, it is important to have a method of measurement as valid and reliable as possible. While direct observation is undoubtedly the most valid and reliable method of measurement, it is also the most expensive means to measure physical restraint use. On the other hand, abstracting data from medical records and interviewing nursing staff have the potential to reduce the cost associated with data collection, but their sensitivity and specificity values need to be demonstrated. In addition, apart from the USA, data sources such as Minimum Data Set (MDS) have not been widely implemented in nursing home facilities throughout the world.

The objective of this study was to determine the sensitivity and specificity of the measurement of physical restraint use reported by members of the nursing staff and reviewed from medical and nursing records among nursing home residents, compared to direct observation. Since underreporting is much more susceptible to be problematic than overreporting, another objective of this study was to compare the sensitivity of the information reported by one nurse with that reported by two nurses or more questioned together. Our research hypothesis was that sensitivity of the interview is highest when the information is collected from more than one nurse.

## Methods

The study was conducted in eight facilities representing a convenience sample of the long-term care facilities in the Quebec City area, Canada. These institutions were carefully selected in order to include a mix of characteristics in size (small and large), geographic location (urban and rural), university affiliation and vocation (units associated with psychiatric or rehabilitation team). Selection was made after discussion with nursing direction of each setting to gather units of different practice such as regular units and specialized units for residents with dementia or severe behavioral problems. Twenty-five subjects were randomly chosen from each unit; if an unit comprised less than 25 residents, all of its residents were included. This study was approved by the ethics committee at Laval University. Data collection took place between January and June 1992.

### Definition of physical restraint

A physical restraint was defined as a mechanical means applied on a resident in order to interfere with his/her mobility, including: vest, waist, wrist or ankle restraints, geriatric chair or wheelchair with fixed tray table, or any other type of locally designed devices [23]. Restrictive siderails, defined as two raised full-length siderails [24], were considered as an intermediate measure and analyzed separately because they are frequently used to prevent bed-related falls during nighttime in long-term care settings [25].

### Physical restraint measurements

Physical restraint use was measured according to three methods: direct observation, interview with members of the nursing staff including licensed practical as well as registered nurses (one or more than one nurse, generally two, questioned together), and review of medical and nursing notes.

### Direct observation

Direct observation of restraints on care units were made independently by two trained research assistants using a pre-tested questionnaire. For practical reasons, observations were made before the chart reviews and the nurses' interviews on three occasions (7h00 AM, 11h00 AM and 3h30 PM) on one day. These specific times were selected as being representative of periods of different nurse staffing, and of overloaded periods during morning and afternoon.

### Interview with nursing staff

In order to reduce the occurrence of an information bias, the nursing staff was blinded to the main objective of the research project. Structured interviews were carried out the day following direct observation by one of the authors (PJD), who was unaware of the observations. Interviews with the nurse in charge of each unit were scheduled, although he/she had the liberty to be represented or assisted by other members of the nursing staff. Physical restraint use on each subject was identified for every hour during the last 24 hours, without knowledge of the times that direct observation was made, by means of a pre-tested questionnaire. The questionnaire covered questions about types of physical restraints (belt, vest, wrist, ankle, fixed tray table, siderails), reasons for use (risk of falls, agitation, wandering, aggressive behaviors, body alignment problems) and the duration including hours and minutes. Other information collected during the interview included: gross cognitive and functional information, risks for falls, history of falls during the last month, agitation, wandering, aggressive behavior and body alignment problems. Cognitive status was evaluated according to five items: recall, speech, and orientation to time, space and people. Three aspects of the functional status were assessed: urinary incontinence, fecal incontinence, and ability to transfer. Respondents could refer to subjects' clinical records at any time during the interview.

### Review of medical files

Restraint use from subjects' medical charts and nursing orders for the last six months was reviewed with a pre-tested questionnaire by a research assistant who was blinded to the observations. Additional information taken into consideration comprised: demographic characteristics, prescriptions for restraints, methods of resident supervision, and psychotropic medications administered in the last 48 hours.

### Statistical analysis

Sociodemographic characteristics of the study sample as well as physical restraint use by methods of data collection were examined using descriptive analysis. Interrater reliability between the two research assistants was tested using the kappa statistic. Direct observation served as the gold standard [26]. To be declared concordant, an observation had to agree with the nurses' interviews on the type of restraint, and on the time of use within one hour. This time frame was set to allow a margin of error of 30 minutes for a reported information and because assessment of restraint use per care unit took an average of another 30 minutes. Each observation was considered as an event independent from one another which may produce a slight overestimation of the precision but no bias. Sensitivity (probability that a person with restraints will be classified as such) and specificity (probability that a person without restraints will be classified as such) values were calculated according to 2-by-2 contingency tables. Differences between methods of measurement were assessed using the phi coefficient. The relationships between potential determinants of restraint use including residents' characteristics and other specific variables reported by nursing staff, and sensitivity values measured by comparing the use reported by nursing staff to direct observation, were examined using chi square tests. Stratification according to these variables allowed to identify specific reasons of underreporting restraint use in the context of a descriptive study.

## Results

Data collection was carried out in 16 nursing units. Of these units, eight depicted skilled nursing home care units, seven nursing home care units, and one long-term care unit within a short-term care hospital. Information was collected for 377 residents with the help of 28 nurses. Residents' age ranged from 32 to 102 years, with a median of 80 years. The sample was 62% female, and median length of stay was 45 months (0 to 720 months). Benzodiazepines and neuroleptics were administered to 35% and 25% of the subjects, respectively.

A total of 6,744 observations over a possibility of 6,786 were made (377 residents by three direct observations and six types of restraints). Prevalence results on physical restraint use according to direct observations (interrater reliability = 92.7%; kappa coefficient = 0.86 (95% confidence interval (CI): 0.73–0.97)), interviews with nursing staff and reviews of clinical records are summarized in Table 1. Fixed tray tables were observed in 23.6% of residents, belts in 12.7% and vests in 4.0% whereas wrist, ankle or other restraints (including locally designed devices, straps or blankets) were used marginally. The nursing staff reported the use of lapboards, belts and vests in 27.6, 17.2 and 5.6% of residents, respectively. Medical and nursing records specified the use of lapboards in 17.2% of residents, the use of belts in 19.4% and the use of vests in 8%. Overall, one third (33.7%) of residents were observed restrained, 32.4% of residents were reported as such by members of the nursing staff, and 38.2% of residents in medical records. Siderails were observed in 62.9% of residents while they were reported by nursing staff in 63.7% of residents, and were mentioned in 72.1% of residents' clinical records.

**Table 1:** Physical restraint use according to a) direct observation, b) interviews with the nursing staff, and c) reviews of medical and nursing records, among 377 nursing home residents

	Physical restraint use					
	Direct observation		Interview with nursing staff		Review of clinical records	

Physical restraint	N	(%)	N	(%)	N	(%)

Fixed tray table	89	(23.6)	104	(27.6)	65	(17.2)
Belt	48	(12.7)	65	(17.2)	73	(19.4)
Vest	15	(4.0)	21	(5.6)	30	(8.0)
Wrist	2	(0.5)	1	(0.3)	2	(0.5)
Ankle	0	(0)	1	(0.3)	0	(0)
Others	3	(0.8)	5	(1.3)	14	(3.7)
Any physical restraints	127	(33.7)	122	(32.4)	144	(38.2)
Siderails	237	(62.9)	240	(63.7)	272	(72.1)

The interview with nursing staff and the review of medical and nursing orders were both highly associated with the observation data (Table 2). The interview of nursing staff showed a somewhat stronger relationship with direct observation compared to the chart review (phi = 0.84 vs. 0.54). Sensitivity and specificity values of the information were highest when data was measured with the assistance of the nursing staff compared to chart reviews. Reported restraint use according to nursing staff (one nurse or more) gave a sensitivity value of 87.4% at a specificity of 93.7%. When data was reviewed from subjects' medical and nursing notes, sensitivity was reduced to 74.8%, and specificity to 86.3%. Restraint use was underreported in 12.6% (16/127) of interviews with nursing staff, and in 25.2% (32/127) of clinical records whereas it was over reported in 4.4% of interviews, and in 19.6% of clinical records.

**Table 2:** Observed physical restraint use compared to restraint use reported a) by interview with the nursing staff, and b) by review of medical and nursing records, among 377 nursing home residents

	Direct observation				Direct observation		
	Yes	No	Total		Yes	No	Total

a) Interview with nursing staff*				b) Review of medical and nursing records†			

Yes	111	11	122	Yes	95	49	144
No	16	239	255	No	32	201	233
Total	127	250	377	Total	127	250	377

* Sensitivity = 87.4%; specificity = 93.7%; phi = 0.84.

† Sensitivity = 74.8%; specificity = 86.3%; phi = 0.54.

Sensitivity values according to specific residents' characteristics and other reported variables are given in Table 3. Increased sensitivity values by 10% or over were observed for perceived risk for falls, agitated behaviors, body alignment problems, aggressive behaviors, urinary incontinence, fecal incontinence, and incapacity to transfer. Sensitivity of the measurement was similar when two or more nurses were interviewed compared to one nurse, although a higher value was noticed when two nurses were questioned (94.1% vs. 85.1%). Significant relationships between perceived risk for falls (*p *= 0.03), agitated behavior (*p *= 0.04), body alignment problems (*p *< 0.001) and aggressive behavior (*p *= 0.01), and reported restraint use by nursing staff were observed. No association was observed for residents' age and sex, number of nurses interviewed, history of falls, wandering problem, disorientation to time, space or people, recall troubles, speech troubles, urinary and fecal incontinence, and ability to transfer.

**Table 3:** Sensitivity values of specific variables reported by nursing staff, among 377 nursing home residents

Variable	N	Sensitivity
		%

Demographic Characteristic		
Sex		
Male	142	86.4
Female	235	88.0
Age (years)		
< 65	41	96.0
65 – 74	83	89.3
75 – 84	127	83.8
> 85	126	83.8
Reported restraint use		
Nurses interviewed		
One nurse	256	85.1
Two or more nurses	121	94.1
Justification for restraint use		
Perceived risk for falls		
Yes	249	91.3
No	127	77.1
History of falls		
Yes	47	92.9
No	327	86.6
Agitated behaviors		
Yes	92	95.6
No	285	82.9
Wandering		
Yes	53	81.8
No	324	87.9
Body alignment problems		
Yes	131	97.4
No	246	72.6
Aggressive behaviors		
Yes	123	97.8
No	254	81.7
Functional characteristics		
Disorientation to space		
Yes	202	89.9
No	169	82.2
Disorientation to time		
Yes	223	88.5
No	141	81.8
Disorientation to people		
Yes	166	91.1
No	208	82.5
Recall troubles		
Yes	220	88.9
No	144	81.6
Speech troubles		
Yes	184	89.0
No	192	84.1
Urinary incontinence		
Yes	249	88.8
No	128	72.7
Fecal incontinence		
Yes	226	89.2
No	151	75.0
Unable to transfer		
Yes	224	89.2
No	153	75.0

## Discussion

The measurement of physical restraint use according to interview with members of the nursing staff and review of medical charts and nursing orders both reflect accurately the reality observed in long-term care setting residents. Our study has also shown that sensitivity and specificity values of the reported measurement are higher than those calculated from medical charts and nursing orders. This phenomenon is not surprising considering that the keeping of medical and nursing orders in nursing homes isn't usually done on a daily basis [27], as opposed to acute care settings.

The current investigation was carried out in units of diverse facilities. The selection of these facilities was intended to allow the participation of subjects and care units of various characteristics as compared to other studies usually designed [28]. The sample of nursing home residents included in this study corresponded well to the physically and cognitively impaired residents generally housing in long-term care institutions.

Limitations of the current study must be taken into account when interpreting these findings. First, data were collected in 1992. Due to the implementation of the OBRA act, it is probable that the prevalence figures given in the current study are overestimations of those that would be observed in 2004. On the other hand, the province of Quebec just recently launched its first comprehensive policy on physical restraint use [23]. Furthermore, the purpose of this study was to compare the sensitivity values of two reporting techniques with direct observation. This comparison should not be affected by the prevalence of physical restraint use. In addition, although a higher proportion of restrained residents might seem more difficult for the nurses to remember as compared to a lower proportion, the nurses didn't show any hesitation when recalling the use of physical restraints as the majority of residents had been living there for a long period of time. Second, we used a convenience sample of long-term care facilities rather than one drawn randomly. We wanted to determine differences and similarities in various practice facilities regarding physical restrain use. The chosen sample provided a relatively broad range of clinical settings. Also, the assessment by nurses was performed the day after direct observation. This time period was chosen in order to reduce recall bias as much as possible, and therefore increase the sensitivity of the reporting technique although this may not be practical in many situations. Another limitation for the interpretation is the use of a descriptive study design. This design is useful to measure the frequency in which a situation occurs or collect data on possible risk factors, but does not allow to infer causal relationships.

It is well known that the prevalence of residents with physical restraints is usually underreported since a social desirability bias tends to affect the validity of the information when the nursing staff has to declare the use of restraints [29]. Despite that restraints are generally applied for safety reasons, nurses nevertheless experience inner struggle when they have to apply them [11,22]. These feelings could influence the nurses' answer when they are interviewed individually and could introduce subsequently a differential misclassification error. In our study, although the sensitivity value improved when interviews were done with two nurses instead of one, the difference was not considered clinically significant. Reported use of physical restraints by two nurses reduced but did not eliminate the presence of an information bias, since the underreporting went down from 14.9 to 5.9%. On the other hand, we do not think that the resulting effect on the prevalence estimates is of consequence. This phenomenon is equally present but to a much lesser extent in the over reporting data since from 6.2% with one nurse, the prevalence of over reported restraint use was reduced to 1.1% when two nurses were interviewed.

Even though other studies have observed an association between residents' characteristics and the risk of being restrained [8,10,17,30,31], these characteristics were not related to the use of physical restraints. Rather, justifications for the use of physical restraints such as disruptive behaviors (e.g. aggressiveness, agitation, and body alignment problems) were associated with their use. For example, it is noteworthy that the risk for falls as perceived by nursing staff was associated with restraint use whereas a history of falls was not. This means that physical restraints were used as preventive devices for a large proportion of subjects, in spite of numerous studies that do not support such practices [13-15,22].

## Conclusions

Compared to review of clinical records, reported physical restraint use by interviewing nursing staff is a simple, efficient, and valid technique of collection of data regarding their use in nursing homes. No severe information bias was observed even though the use of physical restraints may be associated with poor quality of care. This method of measurement appears to be reliable and valid for research purposes. Moreover, our study provides support to the American initiative in regard to the monitoring of several outcomes in nursing homes through nursing staff reports [32-35]. According to our results, interviewing nursing staff is a sensitive and specific method of eliciting information on physical restraint use. Finally, these results have implications for future research in the field. Interviewing nurses on different aspects of medical and nursing care seemed to be a reliable method.

## Competing interests

The authors declare that they have no competing interests.

## Authors' contributions

DL participated in the second line of statistical analyses, and drafted the manuscript. PV drafted parts of the document and contributed to the editing. RV contributed to the editing of the manuscript. PJD served as the Principal Investigator, designed the study, participated and oversaw field activity, revised and edited the manuscript. All authors read and approved the final manuscript.

## Pre-publication history

The pre-publication history for this paper can be accessed here:


